# Influence of Benzo(a)pyrene on Different Epigenetic Processes

**DOI:** 10.3390/ijms222413453

**Published:** 2021-12-15

**Authors:** Bożena Bukowska, Paulina Sicińska

**Affiliations:** Department of Biophysics of Environmental Pollution, Faculty of Biology and Environmental Protection, University of Lodz, Pomorska Str. 141/143, 90-236 Lodz, Poland; paulina.sicinska@biol.uni.lodz.pl

**Keywords:** benzo(a)pyrene, methylation level, lung cancer, smoke, carcinogenicity, histone modifications

## Abstract

Epigenetic changes constitute one of the processes that is involved in the mechanisms of carcinogenicity. They include dysregulation of DNA methylation processes, disruption of post-translational patterns of histone modifications, and changes in the composition and/or organization of chromatin. Benzo(a)pyrene (BaP) influences DNA methylation and, depending on its concentrations, as well as the type of cell, tissue and organism it causes hypomethylation or hypermethylation. Moreover, the exposure to polyaromatic hydrocarbons (PAHs), including BaP in tobacco smoke results in an altered methylation status of the offsprings. Researches have indicated a potential relationship between toxicity of BaP and deregulation of the biotin homeostasis pathway that plays an important role in the process of carcinogenesis. Animal studies have shown that parental-induced BaP toxicity can be passed on to the F1 generation as studied on marine medaka (*Oryzias melastigma*), and the underlying mechanism is likely related to a disturbance in the circadian rhythm. In addition, ancestral exposure of fish to BaP may cause intergenerational osteotoxicity in non-exposed F3 offsprings. Epidemiological studies of lung cancer have indicated that exposure to BaP is associated with changes in methylation levels at 15 CpG; therefore, changes in DNA methylation may be considered as potential mediators of BaP-induced lung cancer. The mechanism of epigenetic changes induced by BaP are mainly due to the formation of CpG-BPDE adducts, between metabolite of BaP—BPDE and CpG, which leads to changes in the level of 5-methylcytosine. BaP also acts through inhibition of DNA methyltransferases activity, as well as by increasing histone deacetylases HDACs, i.e., HDAC2 and HDAC3 activity. The aim of this review is to discuss the mechanism of the epigenetic action of BaP on the basis of the latest publications.

## 1. General Introduction

**Figure 1:**
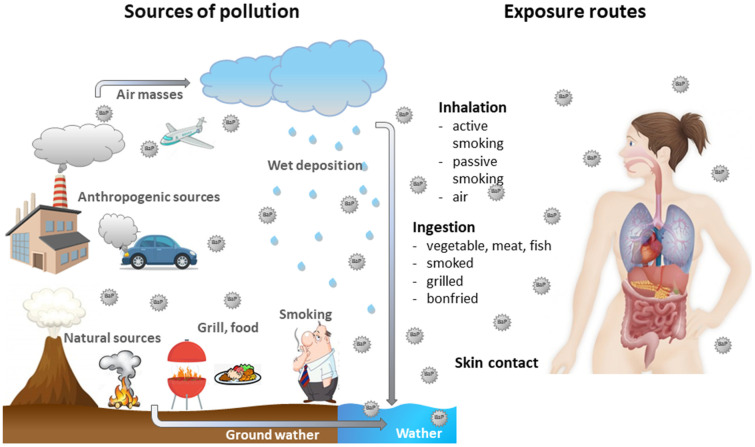
Sources of BaP and its movement in the environment as well as ways of entering the human body.

Benzo[a]pyrene (BaP) is a polycyclic aromatic hydrocarbon (PAH) and is used as an indicator of PAHs. It is primarily produced by burning of fossil fuels, wood and other organic materials. Human exposure to BaP is usually associated with the exposure to other PAHs [1]. BaP is present in cigarette smoke [2], but also in food products, especially in products processed at high temperatures [3,4]. BaP can enter the environment; therefore, it has been found in the atmosphere [5], surface water [6] and soil [7,8]. It has been proven that food, drinking water and the air are the prevalent sources of human exposure to BaP [2,9] (Figure 1).

BaP and especially its metabolite: r-7, t-8-dihydrodiol-t-9,10-oxy-7,8,9,10-tetrahydro-benzo[a]pyrene (BPDE-I), form adducts with DNA (anti-benzo[a]pyrene-7,8-diol-9,10-oxide-DNA adducts) showing mutagenic and carcinogenic effects. BaP is a group I carcinogen [1]. BaP as a component of PAH mixtures increases the risk of cancer of the lung, skin, bladder, breast, kidney, prostate, larynx, hematopoietic system, brain and colon [9,10,11,12,13].

Epigenetic changes are one of the mechanisms responsible for adverse effects of BaP. The main mechanisms of action of BaP involve: (1) creation of stable and depurinating DNA adducts, (2) repetitive redox cycling, which generates reactive oxygen species, (3) activation of the aryl hydrocarbon receptor (AhR), (4) immunosuppression and (5) different epigenetic changes [14]. The epigenetic effect of BaP has been demonstrated in in vitro and in vivo, as well as in epidemiological studies. The ability of carcinogens to disturb epigenetic processes is one of the causes of cancer development.

## 2. Epigenetic Changes and Factors Regulating Them

**Figure 2:**
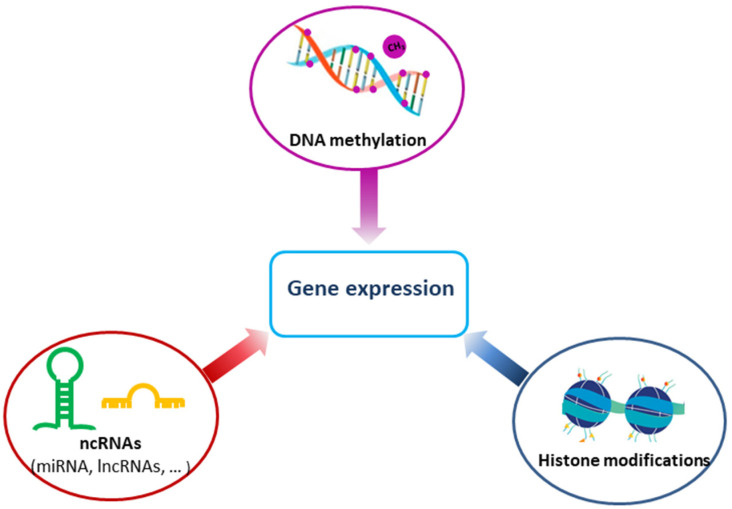
Epigenetic mechanisms contributing to gene regulation, DNA methylation, histone modifications, and noncoding RNAs (ncRNAs).

Epigenetics involves investigations of dynamic and heritable genomic modifications that occur independently on the DNA sequence. These investigations focus on stable modifications of fixed genomes, determining which genes are expressed, and which are silenced. Epigenetic changes require a consistent interaction of various factors with different enzymes and other molecular components. These changes concern hereditary but reversible changes in histones or DNA that regulate activity of a gene, beyond its basic sequence. Epigenetic changes can be observed as abnormal DNA methylation patterns, disturbed patterns of post-translational histone modification, and changes in chromatin composition and/or organization. Epigenetic mechanisms are implicated in the regulation of gene through methylation of DNA, modifications of histones, and through noncoding RNAs (ncRNAs) (Figure 2). Epigenetic mechanisms are also responsible for controlling of gene expression during normal development, while their improper regulation can result in development of human diseases, including cancer. Changes in the epigenome very often accompany development of carcinogenesis and appear already at the stage of initiation of neoplastic transformation, ahead of clinical symptoms characteristic for a specific type of tumor [15].

Base methylation is the most durable, endogenous modification of DNA that involves covalent attachment of a methyl group derived from S-adenosyl-methionine (SAM) to the carbon at the fifth position of the cytosine ring, which is the most common component of the CpG dinucleotide, creating 5-methylcytosine. Cytosines may also be methylated in the CpNpG and CpNpN sequences (N—stands for adenosine, cytidine or thymidine) [16]. The CpG islands are mostly located on the 5 ‘side of the 0.5–4 kbp gene. They are present in about 60% of gene promoter sequences, including almost all promoter sequences of basic metabolism genes and some tissue-specific genes [17]. Methylation reactions are catalyzed by deoxyribonucleic acid methyltransferase, (DNMT). Various types of DNMT, including DNMT1, DNMT2, DNMT3A, DNMT3B are known: Methylation leads to a reduction or silencing of gene expression by modifying the promoter regions of the genes. There are two mechanisms that lead to silencing of gene expression: blocking the binding of transcriptional coactivators and binding of methyl-CpG binding domain (MBD) proteins that bind methyl groups. Methylation also affects the degree of chromatin condensation, which significantly reduces the availability of DNA for transcription factors. In mammalian cells, DNMT1 is responsible for maintaining DNA methylation status by restoring the methylation pattern following DNA replication. On the other hand, DNMT3A and DNMT3B perform de novo methylation during early embryonic development, when the methylation pattern is yet to be established [16].

Cytosine methylation in CpG islands located in promoter regions can lead to the silencing of gene expression at the transcriptional level. The formation of a spherical hindrance, which is a methyl group, causes a loss of affinity of transcription factors for the methylated DNA sequences, which prevents gene expression. Binding of repressors as the proteins that bind to methyl-CpG-binding protein can activate histone deacetylase (HDAC) and/or transcriptional corepressors upon recognition of methylated DNA, which results in silencing of gene expression. As a result, chromatin is strongly condensed, preventing the access of transcription factors to the promoters of given genes [18].

In addition to methylation of cytosines included in the CpG islands located within the promoter sequences of given genes, global DNA methylation is also distinguished. Total genome methylation is usually assessed by determining the ratio of 5-methylcytosine to cytosine in the genomic DNA. Complete hypomethylation of DNA can lead to general genome instability, resulting in increased transcriptional activity [19].

Post-translational modifications of histones, consisting in the covalent attachment/detachment of functional groups to histones, belong to the epigenetic mechanisms contributing to the modification of the chromatin structure. Histone modifications play an important role in maintaining homeostasis between transcriptionally active euchromatin and transcriptionally inactive heterochromatin. Depending on the position of the attached functional group and the type of amino acid residue undergoing modification of a given histone, these processes may lead to activation/repression of gene expression [20].

A number of histone modifications, including acetylation, phosphorylation, methylation, ubiquitilation, sumoylation, ADP-ribosylation and biotylation can be distinguished. Each of the listed histone modifications is catalyzed by a series of enzymes. Histone acetyltransferases (HATs) attach an acetyl group to a lysine residue and are considered to be transcriptional coactivators. In turn, histone deacetylases, which are transcriptional corepressors, remove the acetyl group. Histone methyltransferase (HMT) transfers a methyl group derived from SAM to a lysine or arginine residue, and histone demethylase catalyzes its detachment. Histone methylation can result in both activation and repression of gene expression depending on the level of methylation and the position of the amino acid residue in the protein. Trimethylation of the lysine residue at position 4 in histone H3 (H3K4me3) and trimethylation of the lysine residue at position 36 in histone H3 (H3K36me3) in the region of the promoter sequences of genes are associated with the activation of transcription of these genes. In contrast, trimethylation of the lysine residue at position 9 of histone H3 (H3K9me3) and trimethylation of the lysine residue at position 27 of histone H3 (H3K27me3) in the promoter regions of these genes correlate with the silencing of their expression. On the other hand, phosphorylation of histones by introducing a negative charge and decondensing the chromatin structure allows access to transcription factors and is associated with its activation. Phosphorylation is performed, among others, by cyclin-dependent kinases, mitogen- and stress-activated protein kinase (MSK) and Aurora B kinases [20,21].

MicroRNAs (miRNAs) play an important role in toxicological studies, because they are strong negative regulators of mRNA levels, and therefore they may be responsible for the modulation of important mRNA networks related to harmful effects of chemicals on cellular processes. MiRNAs are one of the key factors involved in the course of epigenetic mechanisms, changes in the level of which have been associated with the toxicity of xenobiotics by modulating gene expression after transcription. New miRNAs that indicate exposure to a given compound, i.e., biomarkers of specific exposure, are constantly discovered [22]. Despite the fact that mRNAs are only 19–25 nucleotides long and are not able to encode any proteins, they influence regulation of gene expression through post-transcriptional modifications. They play the role of regulators of gene expression [23], they combine with mRNA that is complementary to them, preventing translation of mRNA into protein (gene silencing) [24]. MiRNA can influence epigenetic regulators, such as DNA methyltransferases or histone deacetylases. Moreover, about half of miRNA genes are connected with CpG islands. The expression of these genes is therefore dependent on the methylation of DNA; additionally, it is affected by histones’ modifications [23].

A number of chemicals can positively or negatively regulate epigenetic processes, ultimately affecting human health. Phytochemicals and other bioactive compounds present in food can restore global and gene-specific patterns of promoter DNA methylation by reactivating DNA methyltransferases or providing methyl groups. Phytochemicals have been mainly found in fruits, seeds, and vegetables, as well as in dietary supplements. These compounds act as powerful antioxidants and anti-cancer agents. Certain chemicals contained in food, such as catechins, curcumin, genistein, quercetin, and resveratrol, have strong anti-cancer effects by reversing epigenetic changes connected with activation of oncogenes and inactivation of tumor suppressor genes [25,26].

Epigenetic changes are also modulated by environmental factors, making them an intermediary between genes and the environment. The chemicals, such as BaP can adversely modify human epigenetic characteristics, leading to many health disorders. DNA methylation is the most studied epigenetic regulator in relation to environmental exposures and xenobiotic toxicity. DNA methylation has been analyzed in terms of the effect of glyphosate [27] and its metabolite—aminomethylphosphonic acid (AMPA) [28], phosphorus flame retardants [29], aflatoxin B1, bisphenol A, air pollutants, persistent organic pollutants, arsenic, cadmium, chromium, lead, mercury, tobacco smoke, nutritional factors or BaP [30].

In this study, a great deal of evidence has been presented to show that BaP, an environmental pollutant and carcinogen, affects a number of epigenetic processes that can potentially result in various diseases, e.g., lung cancer, development.

## 3. BaP Changes Global and Gene Specific DNA Methylation

To date, numerous studies have shown in detail how the environment influences DNA methylation, leading to altered global as well as specific DNA methylation of a specific gene. These studies have focused on the exposure scenarios during the prenatal period, early life and adulthood [30].

In vitro studies have shown that BaP, a developmental and reproductive carcinogen, is an epigenetic modifier. In the early 1980s, several studies used BaP and its mutagenic metabolite—anti-7β,8α-dihydroxy-9α,10α-epoxy-7,8,9,10-tetrahydrobenzo[a]pyrene (BPDE) to study modulation of DNA methylation in vitro. BPDE was shown to bind to DNA, which resulted in the methylated DNA formation [31] and alteration of DNA methyltransferase (DNMT) [32].

Two studies have described BaP-induced hypo- and hypermethylation in in vitro cell line models [33,34]. Despite the lack of changes in expression of dnmt1, dnmt3a or dnmt13b mRNA, an increased expression of the DNMT1 protein and hypermethylation of promoter of several genes (from a panel of 30 genes analyzed) in immortalized bronchial epithelial cells was observed [35]. After treatment of immortalized bronchial epithelial cells with BPDE, the concentration of cytosine-DNA-1 methyltransferase increased, which was associated with hypermethylation of 5–10 gene promoters, including members of the cadherin gene-family [35]. However, when untransformed cells were treated with BPDE in vitro, no significant changes in methylation status were observed [36].

Studies on the effect of PAHs, and especially BaP, on global DNA methylation are quite limited and contradictory. For example, an in vitro study with TK6 cells exposed to benzo[a]fluoranthene, BaP, and benzo[a]anthracene [37], showed no global DNA methylation changes, while other studies have revealed that exposure to BaP induced global DNA hypomethylation in zebrafish (*Danio rerio*) embryos [38], and global DNA hypermethylation in mouse embryonic fibroblasts [39]. Similarly, primary human bronchial epithelial cell lines (16HBE cells) treated with BaP showed changes in DNA methylation, including hypermethylation and hypomethylation [35,40].

All of the above-mentioned studies have used high concentrations of BaP or BPDE. In contrast, Fang et al. [41] exposed zebrafish embryos to environmentally relevant concentrations of waterborne BaP (24 μg/L for 2.5 to 96 h) and measured both global and gene-specific DNA methylation of five developmentally important genes, namely *VASA*, ras-association domain family member 1 (*RASSF1*), telomerase reverse transcriptase (*TERT*), *C-JUN* and *C-MYCA*. These researchers demonstrated that BaP significantly reduced (by 44.8%) global cytosine methylation and decreased (by 17%) promoter methylation in *VASA a*. As a consequence, *VASA* expression was increased significantly (by 33%). In contrast, the exposure of zebrafish larvae to environmentally relevant concentrations of BaP did not alter methylation of CpG island nor affected gene expression in cancer genes, including ras-association domain family member 1 (*RASSF1*), telomerase reverse transcriptase (tert), *C-JUN*, and *C-MYCA*. Similarly, BaP made no changes in gene expression of *DNMT1* and glycine N-methyltransferase (*GNMT*). Although total DNMT activity was not changed, the activity of the GNMT was moderately increased. Therefore, the authors suggested that BaP behaved as an epigenetic modifier of specific and global DNA methylation in zebrafish larvae.

More recently, Zhao et al. [42] investigated the mechanism of changes in the whole genomic DNA methylation of the Institute of Cancer Research (ICR) mice exposed to BaP. Blood, liver, pancreas, skin, lung, and bladder of IRC mice were removed and analyzed following a 6-h BaP exposure, and total genomic DNA methylation level was determined by high performance liquid chromatography. The results showed the tissue specificity of global DNA methylation, i.e., the total genomic DNA methylation level was significantly decreased in the blood and liver but not in the pancreas, lungs, skin and bladder of the tested animals.

The studies on the effects of paternal exposure to BaP occurring in offsprings and underlying mechanisms are very limited. Zhang et al. [43] found epigenetic changes in BaP exposed rats compared to unexposed rats. Rats in the BaP group were exposed to 0.1 mL/100 g (body weight) by intraperitoneal injection, and the BaP concentration was 0.1 mg/(kg/day) for 60 days. Rats in the control group were injected intraperitoneally with 0.5% of DMSO at 0.1 mL/100 g (body weight). Exposure to BaP resulted in hypomethylation of 3227 genes and hypermethylation of 828 genes. Kyoto Encyclopedia of Genes and Genomes pathway analysis showed that the DMGs were significantly enriched in the Ras and Rap1 signalling pathways, pancreatic secretion and neuroactive ligand-receptor interaction. Moreover, DisGeNET disease spectrum analysis showed that DMGs were associated with infertility and certain genetic diseases [43].

A growing body of human and animal studies report that exposure to BaP causes neurological abnormalities and is also associated with adverse effects, such as tumor formation, immunosuppression, teratogenicity, and hormonal disruptions. Zhang et al. [44] investigated regulatory mechanisms underlying effects of chronic BaP exposure on neurobehavioral performance in mice. The authors treated C57BL mice with various doses of BaP (1.0, 2.5, 6.25 mg/kg) dissolved in olive oil or only with olive oil. The mice were administered with BaP by intraperitoneal injections (volume 50 μL) twice a week for 12 weeks. It was found that mice that received BaP (2.5 mg/kg, 6.25 mg/kg) showed short-term memory deficits and anxiety-like behaviors. These behavioral changes were associated with downregulation of the NR2B gene and concomitant increases in DNA methylation levels in the *NR2B* promoter in two regions of the brain. The authors postulated that chronic exposure to BaP induced an increase in DNA methylation in the promoter of the *NR2B* gene and lowered *NR2B* expression, which may have contributed to neurotoxic effect of this compound, and the related changes in the behavior of tested animals. These results suggested that NR2B susceptibility is a target for environmental toxins in the brain.

### Hypomethylation Induced by Benzo(a)pyrene and the Role of Poly(ADP-ribose) Glycohydrolase Silencing in DNA

Poly(ADP-ribose) glycohydrolase (PARG) is the primary enzyme that catalyzes the hydrolysis of poly(ADP-ribose) and participates in a number of biological processes, including the repair of DNA damage, chromatin dynamics, transcriptional regulation, and cell death. Hung et al. [40] showed that poly(ADPribosyl)ation is involved in maintenance of DNA methylation level and may prevent cancer development induced by BaP.

They used two cell lines: primary human bronchial epithelial cell lines (16HBE cells) and PARG-deficient human bronchial epithelial cell line (shPARG cell) as an in vitro model, and investigated the role of PARG silencing in DNA methylation pattern changed by BaP. They showed that BaP treatment decreased global DNA methylation level in 16HBE cells in a dose-dependent manner, but no dramatic changes were observed in shPARG cells. Further investigation revealed that PARG silencing protected DNA methyltransferases (DNMTs) activity from change by BaP exposure. These results showed an important role for PARG silencing in DNA hypomethylation induced by BaP.

## 4. Epigenome-Wide DNA Methylation and Its Mediation Role in BaP-Associated Lung Cancer Development

During oncogenesis, the epigenome undergoes many changes, including genome-wide DNA methylation decline, regional hypermethylation (especially in CpG promoter islands of tumor suppressor genes), global changes in histone modification markers and alterations in miRNA expression [45].

### Impact of Benzo[a]pyrene-2ʹ-Deoxyguanosine Lesions on Methylation of DNA

DNA damage caused by the binding of carcinogenic 9S,10R-epoxide7R, 8S-diol (BaPDE), a metabolite of BaP, with guanine in CpG dinucleotide sequences may affect DNA methylation, which represents a potential epigenetic mechanism of chemical carcinogenesis. Subach et al. [46] investigated the effect of stereoisomeric adducts (+)- and (−)-trans-anti-BaPN(2)-dG(B(+) and B(−)) on DNA methylation by prokaryotic DNA methyltransferases, such as M.SssI and M.HhaI. These enzymes can recognize sequences like CpG and GCGC, and are capable of transferring methyl group to the C5 atom of cytosine. It has been shown that BaPDE lesions had no effect on M.SssI binding to DNA, but decreased M.HhaI binding. In the majority of cases, residues of BaP decreased methylation efficiency of hemimethylated and unmethylated DNA by M.SssI and M.HhaI.

**Figure 3:**
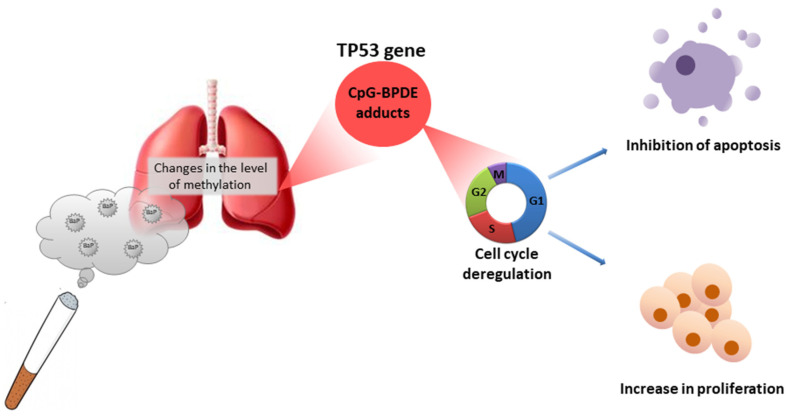
TP53 gene and its role in the exposure to BaP [44,45,46].

*TP53* tumor suppressor plays a special role in cancer development. It is responsible for genomic stability and cellular homeostasis by coordinating various effector processes and pathways, including regulating the cell cycle (e.g., cell cycle arrest in the G1 phase) and inducing apoptosis in any genotoxic stress-induced events during replication. Loss of tumor suppressive activity due to missense mutations in the *TP53* gene, which is particularly prevalent in human tumors, reverses this protective role [47] (Figure 3).

A large proportion of the *TP53* mutations in smokers’ lung cancers (exposed to BaP) are G-to-T transversions, a type of mutation that is rare in lung cancers of non-smokers and in most other cancers. Previous studies have shown that there was an association between the G-to-T transversion hotspots in lung tumors and sites of preferential production of these changes by PAHs in the *TP53* gene. *TP53* codons containing methylated CpG sequences are the preferred adduct targets of the BaP metabolite, BPDE. To assess the role of CpG methylation in mutation induction by BPDE, Yoon et al. [48] analyzed mutagenic changes caused by BPDE in three methylated CpG target genes: supF shuttle vector and cII and lacI transgenes in murine embryonic fibroblasts. Following methylation of the shuttle vector in all CpG sequences, 42% of all G-to-T transversions were at CpG sites compared to 23% in unmethylated DNA. In the cII transgene, which is methylated in CpG sequences in vivo, 56% of BPDE-induced mutations were G-to-T transversions, and 58% of all G-to-T transversions occurred in methylated CpG sequences. In the lacI gene, 68% of BPDE-induced mutations were G-to-T events, and 77% of these events occurred in methylated CpG sequences. Presence of transversion hotspots in methylated CpGs correlated with high levels of BPDE adducts formed at these sites. This situation mirrors the *TP53* gene situation in smokers’ lung cancer, where 51% of G-to-T transversions occurred at the CpG methylated sites (Figure 3).

A very important epidemiological study was conducted by Meng et al. [49]. They performed the first whole epigenome DNA methylation analysis after BaP exposure in two case-control studies of lung cancer in 462 patients. They identified 15 CpGs associated with BPDE-Alb adducts, among which the methylation levels at five loci (*cg06245338*, *cg24256211*, *cg15107887*, *cg02211741*, and *cg04354393* annotated to UBE2O, SAMD4A, ACBD6, DGKZ, and SLFN13, respectively) mediated a separate 38.5%, 29.2%, 41.5%, 47.7%, 56.5%, and a joint 58.2% of the association between BPDE-Alb adducts and lung cancer risk. Compared to the traditional factors, addition of these CpGs exerted improved discriminations for lung cancer with AUC ranging from 0.828 to 0.861. These researchers identified changes in DNA methylation as potential mediators in the formation of lung cancer induced by BaP (Figure 3).

## 5. Prenatal Exposure to PAHs and BaP and Changes in Methylation Levels

BaP is usually determined in high concentration in PAHs mixtures, to which tobacco smokers and workers employed in various industries are particularly exposed. Thus, when assessing the associations between adverse health effects and the exposure to a PAHs mixture, it is justified to relate these relationships indirectly to BaP.

Studies on human cohorts exposed to BaP in tobacco smokers have suggested the associations between BaP exposure and altered DNA methylation status in the offsprings [50,51,52]. Prenatal exposure to BaP and other PAHs have been shown to be associated with reduction in global DNA methylation in adults in the Polish, Chinese and US population [50,53,54,55,56,57].

**Figure 4:**
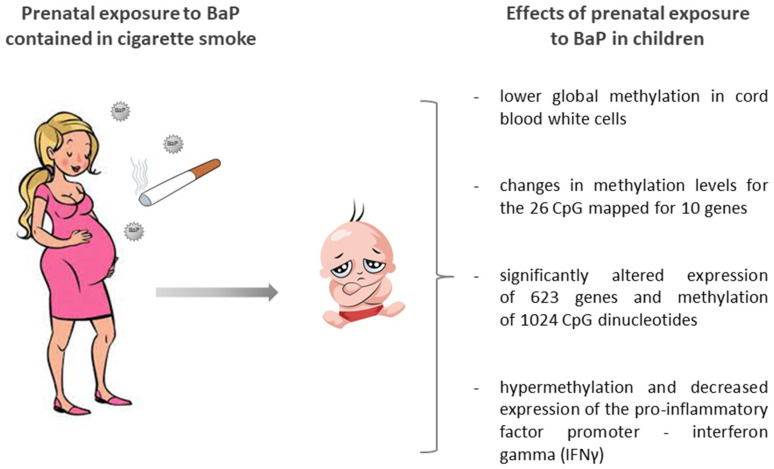
Prenatal exposure to PAHs and BaP and changes in methylation levels [50,51,52,58].

Herbstman et al. [50] examined the effect of prenatal PAHs exposure on fetal umbilical cord blood genomic DNA methylation in a long-term cohort study of non-smoking women (New York, USA) and a relationship between methylation levels and the presence of detectable PAH-DNA adducts. They randomly selected 164 participants from the Columbia Children’s Center for Environmental Health (CCCEH) cohort of 725 women with stored cord blood DNA, half with prenatal PAH exposure levels above and half with exposure below the population median (PAHs, including pyrene, 5.314 ng/m^3^; PAHs, not including pyrene, 2.265 ng/m^3^). They observed that prenatal exposure to PAHs was correlated with lower global methylation of DNA in umbilical cord blood cells, and showed that the levels of global methylation were positively correlated with the occurrence of detectable adducts in cord blood. The authors concluded that prenatal exposure to PAHs caused alterations in the levels of fetal methylation (Figure 4). A similar study was conducted by Joubert et al. [51]. These researchers examined methylation of the entire epigenome in the umbilical cord blood of 1062 newborns whose mothers smoked or not during pregnancy. The results showed that genes characterized by methylation changes were identified in children whose mothers smoked during pregnancy. DNA methylation was demonstrated with statistical significance for the entire epigenome for 26 CpG mapped for 10 genes. Similarly, the study by Suter et al. [52] suggested that frequent perinatal exposure to PAHs (such as maternal smoking) deregulated placental cell DNA methylation in a CpG site specific manner, which correlated with significant changes in gene expression along signature pathways. It was shown that expression of 623 genes and methylation of 1024 CpG dinucleotides were significantly altered among smokers, with 38 CpG showing significant differential methylation (methylation level difference ≥ 10%) (Figure 4).

Maternal factors play an important role in childhood asthma development. Tang et al. [58] assessed a relationship between maternal PAHs exposure and methylation status of the pro-inflammatory factor interferon gamma (*IFNγ*) and anti-inflammatory factor interleukin 4 (*IL4*) promoter. Differentiation of naive CD4+ T cells into proallergic T helper 2 cells induces IL4 expression and inhibits IFNγ expression accompanied by consistent methylation changes in promoters of their genes. The authors assessed the effect of BaP on the methylation status of the *IFNγ* and *IL4* promoters in Jurkat cells and two lung adenocarcinoma cell lines and on their gene expression. They also examined methylation status of the *IFNγ* promoter in umbilical white blood cells from 53 participants (Columbia Center for Children’s Environmental Health cohort). The mother’s exposure to PAHs was assessed by personal air monitoring during pregnancy. In vitro exposure of cellular models to low, non-cytotoxic concentrations (0.1 and 1 nM) of BaP induced increased promoter hypermethylation and decreased *IFNγ* expression, but did not alter the IL4 gene expression. Consistent with these results for cell lines, maternal PAHs exposure was associated with *IFNγ* hypermethylation in the cord blood DNA of the studied children. These findings supported the potential role of epigenetic changes in fetal cell reprogramming through PAHs-induced environmental exposure (Figure 4).

## 6. The Changes in Histone Methylation and Acetylation

Exposure to BaP has been associated with alterations in epigenetic markers that are involved in development of cancer. Biotinidase (BTD) and holocarboxylase synthetase (HCS) are the enzymes responsible for biotinylation homeostasis, while deregulation of this process has been associated to various cancers development. Xia et al. [59] showed that expression of both BTD and HCS was significantly decreased after treatment of human bronchial epithelial cells (16HBE) with BaP. It was revealed that exposure to BaP led to a global loss of DNA methylation, which coincided with a decrease in histone H3 and H4 acetylation level in 16HBE cells. Consistent with decreased histone acetylation, histone deacetylases (HDACs), i.e., HDAC2 and HDAC3 were significantly upregulated in a concentration-dependent manner. Inhibition of DNA methylation or the activity of HDAC, caused that reduction in the activity of BTD and HCS was regulated by means of other epigenetic mechanisms. The authors of this study suggested that BaP by induction of global hypomethylation may cause cancer development. They suggested a relationship between BaP toxicity and biotin homeostasis pathway deregulation in the BaP-related cancer development.

In another study, researchers used human promoter tiling arrays along with chromatin immunoprecipitation to identify changes in histone acetylation and showed that profiles of genome-wide histone H3K9 acetylation were changed in MCF7 breast cancer cells incubated with BaP [60].

### Paternal Exposure to Hydroxylated PAHs Metabolites Significantly Affects the Birth Weight of a Newborn—The Role of Methylation in the H19 Gene

The increase in *H19* gene methylation under the influence of BaP was suggested by Yang et al. [61]. These researchers examined urine collected from 302 men of reproductive age (22–46 years), and collected demographic data using questionnaires. Analysis of a correlation between hydroxylated PAHs (OH-PAHs) levels and methylation levels of imprinting genes showed that the level of OH-PAHs was associated with some CpG sites in *H19, PEG3* and *MEG3*. A negative correlation was found between birth weight and *H19*, namely, each percent increase in *H19* gene methylation (but not Peg3 and Meg3) was significantly associated with a weight reduction of 0.135 g. These results showed that the paternal environmental exposure to PAHs (probably, including BaP) significantly influenced the newborn’s birth weight and that the methylation of the *H19* gene may be involved in the mechanisms underlying this process. This human population study supported previous results from animal studies and suggested that the effects of BaP on the paternal organism caused changes in the offsprings [61].

## 7. Changes in the Level of Various microRNAs as a New Factor in Response to BaP Exposure

Lizarraga et al. [62] identified possible miRNA-mRNA networks as novel interactions at the level of gene expression after exposure to BaP. They analyzed the time-dependent effect on mRNA and microRNA profiles in HepG2 cells. These authors observed changes in miRNA expression in response to BaP, combined with numerous changes at mRNA levels. Using the pathway analysis, the authors identified eight miRNAs that seemed to participate in specific BaP-responsive pathways important for the genotoxicity of this substance. Pathways, included apoptotic signalling, cell cycle arrest, DNA damage response, and DNA damage repair. These researchers also identified miRNA-29b, miRNA-26a-1* and microRNA-122* as important new factors emerging in response to the toxic effects of BaP.

**Figure 5:**
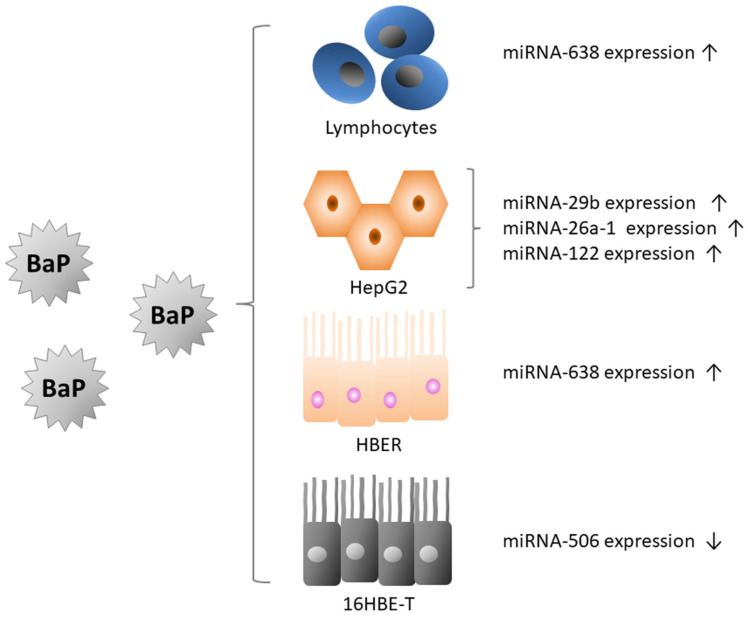
The effect of BaP on various microRNAs levels in different cell types [53,62,63].

Accumulating evidence has suggested that altering miRNA expression, induced by chemical carcinogens, plays an important role in tumor development and progression. Zhao et al. [63] used the anti-BPDE-induced malignant transformation of human bronchial epithelial cell line (16HBE-T) to study mechanisms of human lung carcinogenesis. They found that miR-506 expression was decreased in 16HBE-T compared to normal human bronchial epithelial cells (16HBE). Restoration of miR-506 in 16HBE-T cells led to a decrease in cell proliferation, and a cell cycle arrest in the G0/G1 phase. These researchers showed that miR-506 acted as an anti-oncogenic miRNA (anti-oncomir) in malignantly transformed bronchial cells (Figure 5).

Identification of aberrant miRNA expression during chemical transformation of cells induced by carcinogens will contribute to a better understanding of the important role of miRNA in cancer development. To investigate whether abnormal miRNA expression could be used as a biomarker of xenobiotic exposure in cancer risk assessment, Li et al. [64] analyzed miRNA expression profiles of human bronchial epithelial cells expressing H-Ras allele (HBER) oncogenes at different stages of BaP-induced transformation. These researchers showed that 12 miRNAs were differentially expressed in HBER cells at both the pre-transformed and transformed stages. The presence of differentially expressed miRNAs was demonstrated in transformed cells and tested in 50 primary human non-small cell lung cancer (NSCLC) tissue pairs. Among the miRNAs examined, upregulation of miR-638 was found in 68% of NSCLC tissues. It was also shown that miR-638 expression in HBER cells increased after treatment with BaP in a concentration-dependent manner. Expression of miR-638 was also tested in peripheral lymphocytes of 86 female workers exposed to PAHs. It was found that the average level of miR-638 expression in peripheral lymphocytes of the tested workers increased by 72% compared to the control group. MiR-638 levels were positively correlated with the urinary 1-hydroxypyrene concentration and with PAHs levels. Overexpression of miR-638 aggravated the BaP-induced damage to cell DNA, which could contribute to the suppression of the breast cancer 1 (BRCA1) gene, one of the miR-638 target genes. The authors postulated that miR-638 was involved in BaP-induced carcinogenesis by targeting BRCA1 (Figure 5).

## 8. The Intergenerational Toxic Effects on BaP Exposure Via Interference of the Circadian Rhythm

The circadian rhythm is a transcription-translation feedback oscillator loop, with a cycle of peaks and troughs in gene expression over 24 h. The circadian clock is involved in regulating life processes, such as sleep, metabolism, reproduction, development and immunity. It has been shown that circadian clock circuitry plays a role in the regulation of innate immune function, as well as in adaptive immune and allergic responses [65,66].

BaP is toxic to marine animals and their offsprings, but the underlying intergenerational immunotoxic mechanism is not clearly understood. Yin et al. [67] examined offsprings of marine medaka (*Oryzias melastigma*), whose parents were treated with BaP at 0.5 mg/L, and found disturbances in circadian oscillations and severe DNA damage. Many clock-related genes, such as per1 were significantly modulated in the offsprings. It was found that both *PER1* and *TP53* were significantly inhibited, which changed cell cycle progression and inhibited DNA repair, thus possibly resulted in increased offsprings mortality. Hypermethylation of the *PER1* promoter and abnormal levels of N6-methyl adenosine (m6A) suggested that the underlying mechanism was possibly related to epigenetic modification. F1 larvae from parents exposed to BaP were more sensitive to this substance, which was due to stronger expression of genes responsible for immunological and metabolic processes. Moreover, after paternal exposure to BaP, offsprings showed more severe DNA damage and a higher degree of hypermethylation than offsprings after maternal exposure. Overall, toxic effects of BaP on parents could have been passed on to the F1 generation, and the underlying mechanism was likely related to a characteristic disturbance of the circadian rhythm [67].

## 9. BaP Osteotoxicity and the Regulatory Roles of Epigenetic Factors; Intergenerational Osteotoxicity in Non-Exposed F3 Generation

Recent studies have shown that ancestral exposure to BaP could cause intergenerational osteotoxicity in non-exposed F3 offsprings. Consequences of environmental contamination with BaP/PAHs, can therefore be serious and require reassessment. Mo et al. [68] in their review postulated that transgenerational inheritance of osteotoxicity in fish, caused by the exposure of ancestors to BaP, was mediated by epigenetically dysregulated processes. These researchers proposed two possible epigenetic mechanisms: (i) bone miRNAs are dysregulated via altered DNA methylation and/or histone modifications, affecting target gene expression/activity; (ii) dysregulation of bone genes through altered DNA methylation and/or histone modifications.

## 10. Long-Term Exposure to BaP Inhibits Expression of *ERα*, *CYP19a* and *VTG1* Genes and Is Toxic to Embryos and Sex Differentiation

BaP can cause endocrine disruptions in organisms. Sun et al. [69] evaluated the effect of BaP exposure on marine medaka by assessing embryonic toxicity and analysis of reproductive genes (*ERα*, *CYP19a*, and *VTG1*) to predict sexual differentiation of tested animals. The results showed that high BaP concentrations (200 μg/L) significantly delayed the hatching time of embryos. Moreover, medium/high concentrations of BaP (20 μg/L and 200 μg/L) prolonged the time of sexual maturity of marine medaka. The relative expression of the ERα, cyp19a and vtg1 genes was measured at 5 days post-hatching of embryos. They showed that BaP significantly inhibited expression of genes associated with development of female fish. Consequently, after BaP exposure, there were more males in the offsprings generation. Therefore, these researchers showed that BaP could have delayed the hatching time of the embryos and extended the time of sexual maturity in tested fish. BaP has also antiestrogenic and androgenic activity, and therefore can significantly inhibit the expression of *ER*α, *CYP19a* and *VTG1* genes, which are associated with female reproductive development and higher production of male offsprings by marine medaka parents exposed to this compound.

## 11. Exposure to BaP in Mixture of PAHs Leads to Changes in DNA Modulation and RNA (hydroxy)methylation

A study by Duca, et al. [70] focused on epigenetic changes caused by exposure to a mixture of PAHs, including BaP. Female Long Evans rats were exposed to Priority 16 PAHs (US-EPA) 3 times per week for a period of 90 days. Liver samples were used to assess the (hydroxy)methylation status of genomic DNA/RNA along with reduced and oxidized forms of glutathione. The results of this research revealed that exposure to PAHs mixture caused alterations in reduced glutathione (GSH) level and induced (hydroxy)methylation of DNA and RNA, along with DNA-PAHs adducts formation. Moreover, non-monotonic associations of the response between PAHs concentration, GSH level and DNA (hydroxy)methylation level were shown at environmentally relevant doses of studied compounds.

## 12. Summary

BaP is an indicator for the entire group of PAHs. BaP is mainly produced as a by-product in combustion of fossil fuels and other materials, and it is also formed as a result of other technological processes. This compound contaminates the environment and is commonly found in the atmosphere, water and soil. BaP and its metabolites are used as biomarkers of PAHs exposure both in the general population and in workers occupationally exposed to this substance. BaP enters the human organisms with consumed food, water and inhaled air. The exposure to BaP concerns numerous occupational groups. It is highly toxic, and is a group I carcinogen. It induces a number of cancers, including malignancies of the respiratory and urinary systems. It exhibits toxicity by various mechanisms, including epigenetic processes. BaP exhibits epigenotoxicity (Table 1) by disrupting the methylation processes both of the entire epigenome and of promoters of individual genes. It also affects expression of histones and triggers various miRNAs expression.

## 13. Conclusions

1. Depending on the concentration, type of cell and organism, BaP disrupts DNA methylation processes upstream or downstream, and epigenetic effects are then passed on to the offsprings even in the third generation.
2. Offsprings paternally exposed to BaP, show more severe DNA damage and a higher degree of DNA hypermethylation than offsprings maternally exposed (e.g., fish studies).
3. Epigenetic abnormalities in lung cells exposed to BaP may lead to cancer development.
4. BaP exhibits epigenotoxicity by disrupting methylation processes both of the entire epigenome and of promoters of individual genes.
5. This compound affects expression of histones and triggers expression of various miRNAs that dysregulate gene expression in cell.
6. Epigenetic changes in response to BaP exposure are mainly due to the formation of CpG-BPDE adducts, and are associated with inhibition of DNA methyltransferases activity and increased histone deacetylases activity.

## Figures and Tables

**Table 1:** Potential epigenetic effects induced by BaP and its metabolite BPDE.

Type of Epigenetic Change	Observed Effects		References
Global methylation	Increased global metylation:	Mouse embryonic fibroblast cells	[39]
		Human bronchial epithelial cells (16HBE cells)	[35]
		Mouse embryonic fibroblast cells	[39]
		Normal human bronchial epithelial cells (NHBE)	[31]
	Decreased global methylation:	Human bronchial epithelial cell line (16HBE)	[40]
		Zebrafish embryos	[38]
		Zebrafish embryos	[41]
		IRC mice	[42]
		Children, whose mothers smoked during pregnancy	[51]
	No changes in global methylation:	Human cells	[36]
		TK6 cells	[37]
Single gene promoters methylation and gene expression	Increased methylation in gene promoter and decreased expression in gene promoter	*IFNγ*—Jurkat cells and 53 women and childrenfrom the Columbia cohort	[58]
		*NR2B*—C57BL mice	[44]
		*H19*—302 reproductive-aged males (22–46 years old)	[61]
		*PER1*—medaka fish *Oryzias melastigma*	[67]
		Biotinidase and holocarboxylase synthetaseHuman Bronchial Epithelial Cells (16HBE)	[59]
		828 hypermethylated genes in rats	[43]
	Decreased methylation in gene promoter	3 227 hypomethylated genes in rats	[43]
	Decreased gene expression	*ERα*, *CYP19a* and *VTG1* expression—*Oryzias Melastigma*	[69]
Histone modifications	Reduction in acetylation levels on H3 and H4 histones	Human bronchial epithelial cells (16HBE)	[59]
	Increase in histone deacetylases HDAC2 and HDAC3	Human bronchial epithelial cells (16HBE)	[59]
	Changes in H3K9 histone acetylation	Breast cancer cells (MCF7)	[60]
Changes in miRNA level	Decreased expression	miR-506 in cancer cells (16HBE-T)	[63]
	Increased expression	miR-638 in breast cancer (BRCA1)	[64]
		mikroRNA-29b, mikroRNA-26a-1* i mikroRNA-122* (HepG2 cells)	[62]

## Data Availability

Not applicable.
